# 500+ FACs Perovskite Solar Cells under Long-Term Stability Testing

**DOI:** 10.1021/acsami.5c24756

**Published:** 2026-01-07

**Authors:** Žan Ajdič, Fernando Solorio Soto, Marko Remec, Miha Kikelj, Arslan Ali, Boštjan Glažar, Gašper Matič, Kristijan Brecl, Marko Jankovec, Marko Topič, Marko Jošt

**Affiliations:** † Faculty of Electrical Engineering, 37663 University of Ljubljana, Tržaška 25, 1000 Ljubljana, Slovenia; ‡ 28340 Helmholtz-Zentrum Berlin für Materialien und Energie, 12489 Berlin, Germany

**Keywords:** perovskite solar cells, long-term stability, maximum power point tracking, moisture and light degradation, voltage bias degradation, photoluminescence

## Abstract

The long-term stability of more than 500 FACs perovskite solar cells has been systematically investigated under various conditions. We first analyze resilience to moisture and show that with the 30 nm Al_2_O_3_ capping, we can perform long-term tests in air and eliminate moisture-related degradation. In the long-term MPP tracking tests, we then confirm that light is the driving degradation contributor by performing cyclic tests and testing under different light intensities. Visual changes of the perovskite absorber during the testing and spatial and spectral photoluminescence measurements reveal that phase segregation and the perovskite/C_60_ interface are the main culprits for degradation, while the perovskite degrades faster in electrically inactive areas. We thus show that by removing bromide ions from the FACs composition, cell stability, evaluated by the *t*_80_ lifetime, can improve 5-fold in the best case and that there is a linear correlation between *t*_80_ time and bias voltage during stability tracking. By testing a large number of samples (>500), we show with statistical relevance that long-term stability measurements show significantly higher spread (both batch-to-batch and intrabatch) than *J*–*V* measurements.

## Graphical abstract

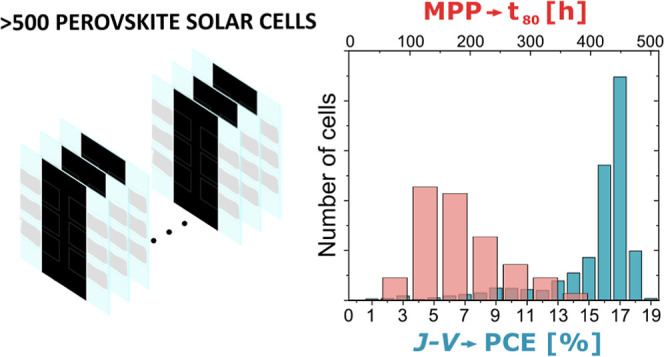

## Introduction

As a relatively new technology, perovskite solar cells (PSCs) already offer an excellent power conversion efficiency (PCE) of 27% under standard test conditions (STC), similar to conventional silicon cells.^1,2^ On the other hand, their long-term stability is still the main drawback preventing perovskites from entering the market, making stability the main focus of research in this emerging technology. Many environmental factors can accelerate the degradation of PSCs and thus shorten their lifetime. The main culprits are humidity, elevated temperature, light, and oxygen.^3–5^ Various methods have already been tested to prevent their influence on the stability. The degradation by moisture and oxygen can be efficiently reduced by encapsulation of devices.^6,7^ In the best case, the encapsulated cells performed outdoors for over a year without noticeable long-term degradation.^8^ Since the encapsulation of many devices is a time and equipment-demanding process, other, simpler ways, such as capping, can be used.^9,10^ In contrast, eliminating light and temperature stress is impossible in a normal solar cell operation. Their induced degradation is inevitable during in-field operation and therefore requires detailed investigation.

The main method to investigate operational stability of PSCs is long-term maximum power point (MPP) tracking,^11^ typically under constant illumination. Impressive results have been achieved, with the record lifetimes now exceeding 1000 h of operation^12–14^ before the power drops below 80% of the initial one (*t*_80_). Still, many reports show *t*_80_ times in the range of a few hundred hours, strongly influenced by the instability of the perovskite absorber and its interfaces with charge-selective layers.^15^ The main culprit is the perovskite’s ionic structure with mobile ions and cations. Both halide anions (I^–^ and Br^–^) migrate under the influence of light and electric bias.^16,17^ This can cause phase segregation, which limits performance,^18^ and reactions with the electrode.^19^ Similarly, electrode materials, gold, copper, or silver, can migrate into the perovskite absorber, causing degradation.^19,20^

In a typical p–i–n cell architecture, the electron transport layer C_60_ and its interface with the perovskite have been shown to be critical factors that accelerate cell degradation and limit its efficiency.^21–23^ The overall performance can be improved with different treatments of this interface^12,24^ or by omitting the C_60_.^13^ On the other hand, with the rise of self-assembly molecules, the HTL/perovskite seems less of an issue.^25–27^

Despite significant progress in improving stability performances, often only the stability of the champion device is reported, with only a few reports showing stability results of more than a couple of devices.^28,29^ This does not reveal any information about the reproducibility of the results or batch-to-batch variations. Numerous devices have to be shown to confirm the effects and reproducibility with statistical relevance, as has become the norm for the PCE.

In this article, we thoroughly investigate the long-term stability of more than 500 FACs perovskite solar cells. We have systematically performed several experiments to understand the effect of moisture and, especially, light on the degradation of FACs PSCs. The first was evaluated by damp-heat and water tests, and the latter was evaluated by using in-house developed long-term MPP tracking and photoluminescence (PL) measurement setups. We show how to reliably test perovskite solar cells in air, determine the main causes of degradation, and analyze intrabatch and batch-to-batch variations. With more than 500 tested devices, this is to the best of our knowledge from the literature one of the largest studies on perovskite solar cell stability and its reproducibility.

## Results

In this paper, we utilized a p–i–n architecture with MeO-2PACz as a hole-selective contact layer and C_60_ and SnO_2_ as electron-selective contacts. The FACs perovskite used has a composition FA_0.83_Cs_0.17_Pb­(I_0.83_Br_0.17_)_3_ with a bandgap of 1.64 eV, and the devices are capped with a 30 nm Al_2_O_3_ layer unless stated differently. The full solar cell layer stack is as follows: glass|ITO|MeO-2PACz|perovskite|C_60_|SnO_2_|Cu|Al_2_O_3_. Each substrate contains 6 devices with an active area of approximately 0.17 cm^2^ each. The fabricated devices with capping show good and reproducible PCE with a median of 16.4% from more than 1200 cells and the best cells at around 18% (Figure 1). This PCE is reached with a median *V*_OC_ of 1.09 V and a fill factor (FF) of 74.5% (Figure S1).

**Figure 1:**
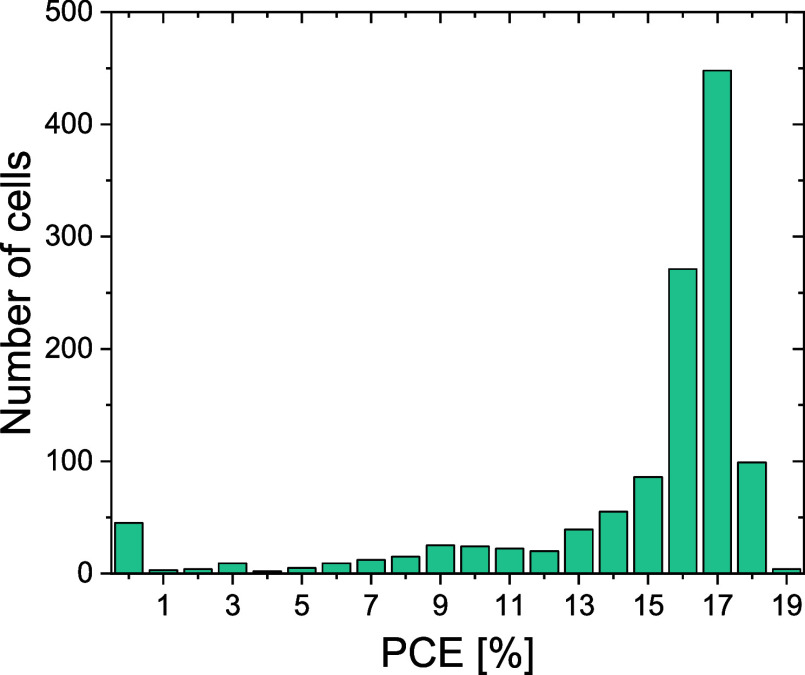
PCE distribution of reference FACs perovskite solar cells, fabricated during multiple batches, and their stability tested in this paper. Cells with a PCE below 14% have mostly been eliminated from the stability testing results.

All MPP tracking tests were carried out in air over a period of more than one year, during which the indoor relative humidity varied from 10% in the winter to 60% in the summer. To ensure reliable and reproducible results of long-term MPP tracking despite varying humidity, we have protected our devices with a 30 nm thick Al_2_O_3_ layer deposited by atomic layer deposition (ALD). This has significantly improved device stability as reported in our previous work^30^ and enabled long-term stability testing in air without the need for encapsulation. In the very best case, these devices can survive more than 1000 h with little degradation (Figure S2a). However, as will be shown later, in most of the tests, the devices have degraded below 80% of their initial performance within approximately 200 h of MPP tracking under continuous 1-sun irradiance. This performance loss raised the question of what the main stressor behind the observed degradation is: moisture or light? And what is the role of sample-to-sample variation?

Since eliminating moisture stress is preferable to eliminating light in the case of solar cells, additional printed circuit board (PCB) spray (Plastik 70-Kontakt Chemie) was applied by hand for further protection. The selected PCB spray is typically used to protect PCBs against moisture and contains PMMA (poly­(methyl methacrylate)), ethyl, and butyl acetate.^31–34^ Applying a spray coating is a fast, simple, and low-cost protection method that has been proven effective for protecting PCBs. However, due to a manual application of spray, its thickness cannot be precisely controlled, making it hard to repeat and control, which is the complete opposite of the ALD. To check the suitability of the moisture-protecting layers (Al_2_O_3_ capping and PCB spray), we have performed water and damp-heat tests and evaluated the performance of films and solar cells by *J*–*V* and PL quantum yield (PLQY) measurements.

The moisture (water and damp-heat) experiments are described in detail in Supporting Information Note 1. In short, devices with capping and spray have survived more than 2 h dipped in water without degradation, while the capped device survived damp-heat testing at 25 and 50 °C and 60% rel. humidity with little degradation. Thus, moisture tests have shown that capping the devices provides sufficient resilience to humidity/water ingress, so we can now investigate the long-term stability of our perovskite solar cells under illumination in air. Testing was performed with a white LED (WLED) setup developed in-house (Figure S2b), which enables simultaneous testing of up to 24 substrates (144 cells) per setup under illumination of white LEDs and different operating points. Here, the tests were performed with devices operating in MPP at a 1 sun equivalent light intensity and a 25 °C cell temperature unless stated otherwise. As a figure of merit for stability, we use the *t*_80_ time, the elapsed time until the performance (PCE) drops below 80% of the initial value. Devices, for which we detected contact issues or other technical problems during the testing, were eliminated from further analysis. With the WLED, we have tested in total more than 500 devices. Such a large number of tested devices provides statistical relevance to our results, going beyond plotting only a champion device.

We started with testing whether the additional moisture protection of the PCB spray introduces any improvements to the long-term stability of devices. For this experiment, we capped all of the fabricated cells and sprayed half of them. The additional spray protection did not affect the initial PCE (Figure S7). Two long-term tests were performed with similar results: the additional spray coating did not noticeably change the long-term stability. Both groups of cells exhibited an average *t*_80_ of around 200 h in the first experiment and around 150 h in the second one (Figure 2a), indicating batch-to-batch variation. The results also showed some spread of data, ranging from below 100 h to above 400 h in both experiments, pointing toward intrabatch variations. This further highlights the need to report stability results of several devices and not just the champion device. Figure S8 shows the time dynamics of *P*_MPP_, *J*_MPP_, and *V*_MPP_ for selected cells from this test. Despite different rates of degradation, in each cell, the degradation is first observable in reduced *J*_MPP_, with *V*_MPP_ following much later. We also examined the dependence of *t*_80_ on the initial PCE but saw no clear correlation between the two parameters (Figure 2b). Since cells with a combination of both capping layers performed best in the water test and reached similar *t*_80_ in the MPPT experiment, we hypothesize that the degradation in our long-term stability tests mostly happens due to light and not moisture. We have thus performed all further tests with capping only.

**Figure 2:**
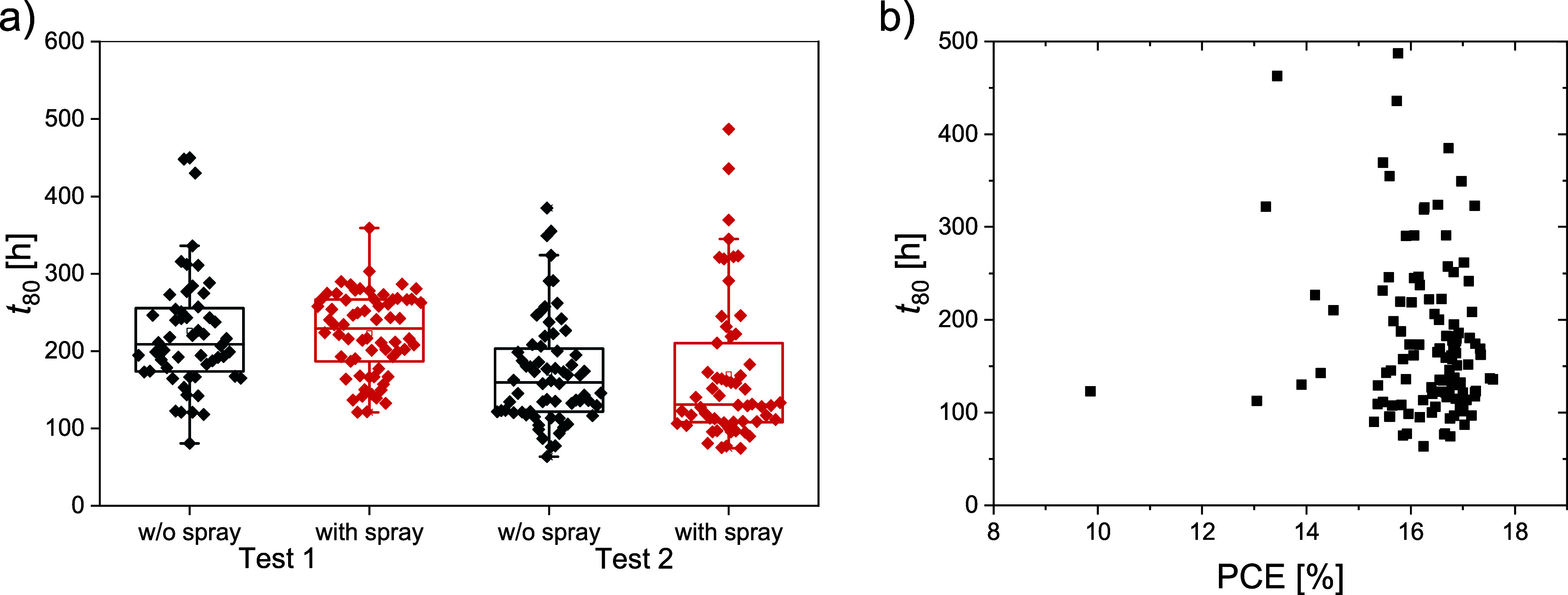
(a) Comparison of long-term stability of capped cells without and with PCB spray. Two experiments were conducted, first in September 2024 and second in December 2024. (b) Dependence of *t*_80_ on the initial PCE. We do not observe any clear correlation.

This hypothesis was further confirmed by altering the illumination conditions in two separate tests. First, in a cycling illumination experiment, we repeatedly exposed cells to 12 h of light and 12 h of dark. Simultaneously, we also ran a continuous illumination test for comparison. From the *t*_80_ results, we observe approximately doubled lifetime (200 to 400 h) for the cells tested in the cyclic illumination regime (Figure 3a). This again suggests light-induced degradation as the main degradation mechanism and not moisture, since the cumulative exposure time to light by the time they reach *t*_80_ is the same for both groups of cells (200 h). Furthermore, the experiment indicates that (at least in this case) there is little recovery due to the dark part of the cycle, contrary to what has been shown before.^35^ The selected MPP tracks for both conditions are shown in Figure S9.

**Figure 3:**
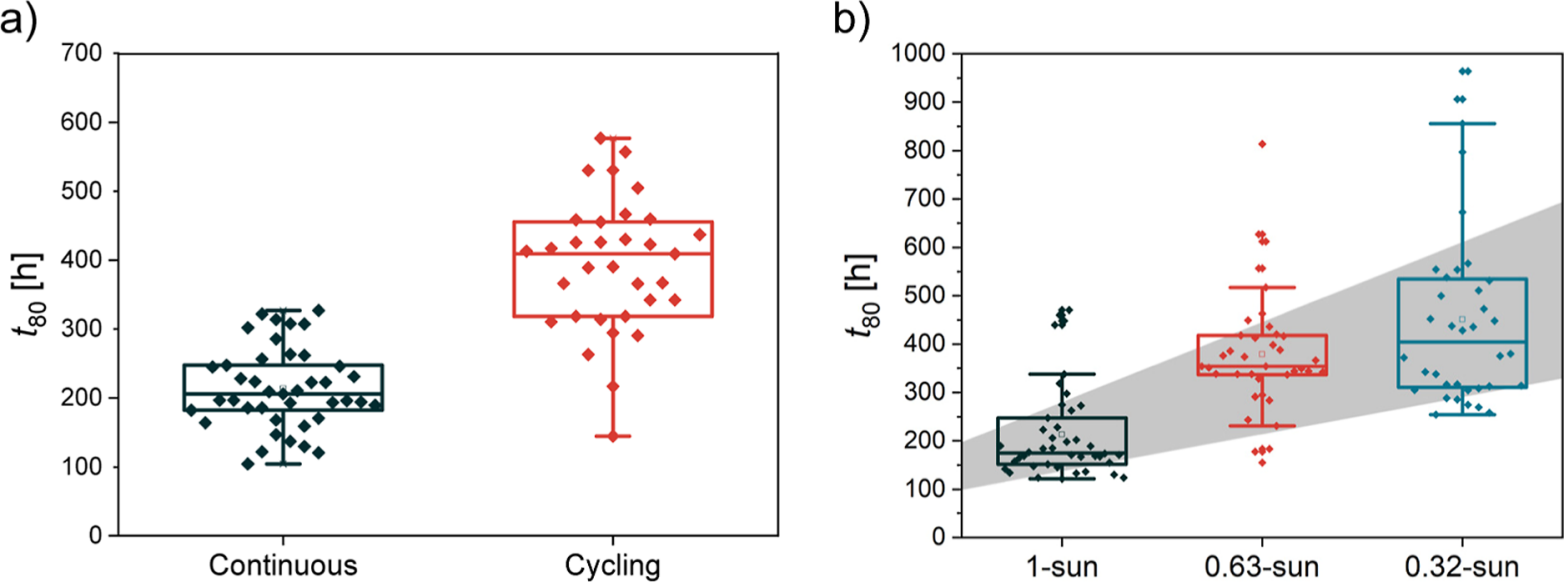
(a) *t*_80_ of solar cells exposed to continuous illumination and cyclic illumination (12 h on/12 h off). For the latter, the lifetime is approximately doubled, which corresponds to the same illumination time of about 200 h in both cases. (b) Dependence of *t*_80_ on the illumination intensity. Solar cells, fabricated in three batches, were covered with homogeneous ND filters that transmit 63% (OD02) and 32% (OD05) of light, respectively. Figure S8 shows the results of each batch.

Second, the cells were exposed to different light intensities, which was achieved by covering some of the samples with a neutral-density (ND) filter to reduce the actual light intensity from 1-sun to 0.63 (OD02) and 0.32 (OD05) sun, respectively. Consequently, we tested 3 different groups of cells in the same setup at the same light intensity (before the filters) at the same time, providing the same ambient temperature and relative humidity. As expected, cells exposed to the lowest light intensity exhibited the longest *t*_80_ times (around 400 h), while reference cells (1-sun, no filter) had the shortest *t*_80_ (below 200 h), as shown in Figure 3b. Nevertheless, here, we did not observe a linear trend with the illumination intensity, like in the cyclic experiment. Instead, with an illumination of 0.32-sun, the *t*_80_ has doubled and not tripled. Possible reasons for that are intrabatch and batch-to-batch variations. Cells for this test were fabricated over 3 different batches within one month, and stability, *t*_80_-wise, each batch performed differently. At the same time, even the cells on different substrates from the same batch and same illumination can yield completely different *t*_80_ (see Figure S10 and discussion at the end of the Results section for batch-to-batch comparison). This observation highlights the need to show results of multiple devices when discussing the stability of perovskite solar cells, since the consistency and reproducibility of stability results vary much more than their PCE results.

Valuable information during long-term testing was also provided by observing the visual changes of solar cells over time. Examples of possible visual outcomes at the end of the test are shown in Figure 4a: (i) the copper electrode has disappeared, (ii) degradation starts in the middle inactive area, exhibiting yellow or (iii) orange color; (iv) degradation starts in the inactive area between the devices under the copper electrode, while sometimes, it also starts (v) around the active area. Finally, (vi) ITO pattern lines often become clearly visible, which could have an impact on (mini)­module lifetime. Finding the root causes of all these different degradation mechanisms will be part of our future research; here, we will focus only on two main cases: (a) in general, the degradation is the strongest in the nonactive areas in between cells, with or without a copper electrode, where the perovskite is in an open-circuit (OC) condition throughout the devices’ lifetime. (b) PL measurements in those critical areas show a very high signal but a strong red shift of the spectra (Figure 4b), most likely caused by phase segregation.

**Figure 4:**
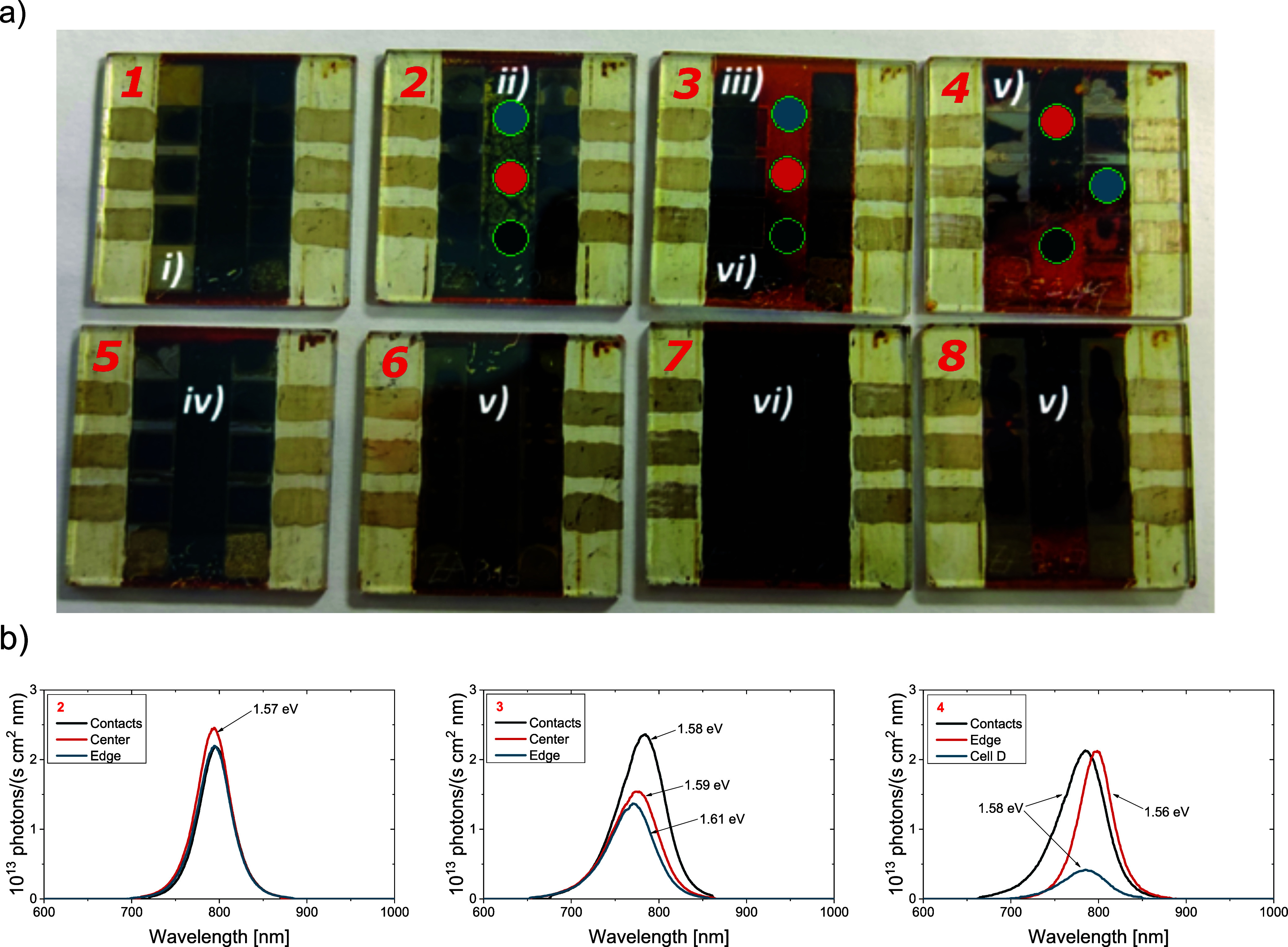
(a) Photo showing visual degradation of cells after an MPP tracking test. Different modes of degradation can be observed, as indicated by the roman numbers and described in the text. Colored circles indicate the position of the PL measurement, and corresponding color lines are plotted in (b).

To observe temporal changes in the PL, we performed long-term spatially resolved PL tracking. Samples were exposed to continuous illumination of blue LEDs (470 nm) with an equivalent intensity of 1-sun, kept at an OC condition, and continuously monitored with a camera, providing spatial information about the PL intensity. Spectral PL information was obtained once a day with a PLQY setup. To determine possible critical layers and interfaces, we fabricated different layer stacks starting with only the perovskite and then layer-by-layer building up to the full stack. In Figure 5, PL images of the initial and the final condition are shown. At the beginning, all films exhibited uniform PL intensity over the whole sample area of 6.25 cm^2^. The PLQY is the highest when only the perovskite is deposited (with or without Al_2_O_3_ capping) and generally decreases with additional transport layers, as is also true for quasi-Fermi-level splitting (QFLS). The initial bandgap was around 1.64 eV for all samples. In the final picture taken after 2 weeks (Figure 5b), clear inhomogeneities appear in the bottom six samples, where C_60_ is deposited on top of the perovskite. Since the PL of samples without C_60_ remains uniform, this indicates a negative impact of C_60_ and its interface with the perovskite layer, causing cell degradation. This is, however, not clear from the PLQY measurements alone, since all the samples show a similar bandgap evolution from the initial values around 1.64 eV to the final values between 1.56 and 1.58 eV (Figure 5c) already after a couple of days of testing and a strong PL intensity increase (Figure S11). Interestingly, samples with ITO experience this transition 2 days later than the samples without the ITO, possibly due to the role of the density of charge carriers and their extraction on phase segregation.^26^ The drastic increase in PLQY with time (4-fold in the case of the bare perovskite) partially compensates for the drop in bandgap, keeping the QFLS relatively stable during the experiment (Figure S12). The same degradation at the perovskite/C_60_ interface also happens for other perovskite compositions presented later in the text (FACsI and “triple cation”, Figure S13), agreeing with previous reports showing improvements in perovskite solar cell stability without C_60_.^13^ Following this, we identify the perovskite/C_60_ interface as the key troublemaker not only for reaching high PCE but also for long-term stability.

**Figure 5:**
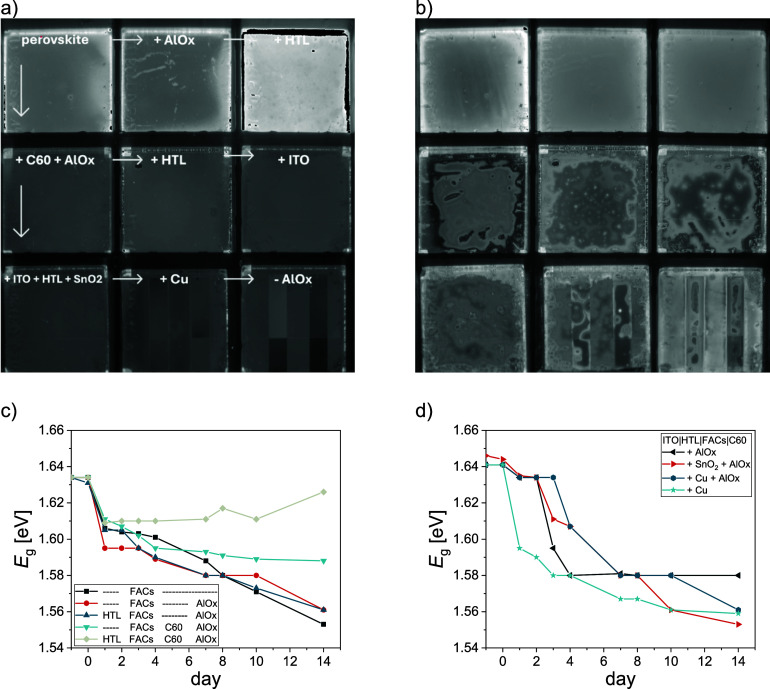
Spatially resolved PL of nine different layer stacks under blue LED light was continuously tracked with a camera. The individual samples are labeled in the photo, starting from only the perovskite without capping, to the whole stack. (a) Photo of PL response of fresh films. (b) Photo of PL response of aged films after 2 weeks. Layer stacks containing C_60_ (bottom two rows) degraded much more than films without it (first row), as indicated by the severe inhomogeneity of the films. Integrated counts during the tracking for each sample are shown in Figure S11. (c,d) Bandgap measurements during the experiment of samples (c) without ITO and (d) with ITO measured with a spectrally resolved PL system. MeO-2PACz was used as the HTL and FA_0.83_Cs_0.17_Pb­(I_0.83_Br_0.17_)_3_ as the perovskite composition (FACs). Corresponding measurements of PLQY and QFLS are shown in Figure S12.

The measured PL of the degraded cells shows a similar bandgap shift (red shift >50 meV) as in the damp-heat experiment. This again indicates phase segregation and emphasizes the need to prevent it as well as to monitor it by regularly acquiring PL spectra of devices. To analyze its effect on stability, we fabricated PSCs with three different compositions: reference FACs with an iodide to bromide ratio of 83:17 (FACsIBr), FACs without bromide (FACsI), and “triple cation” (3C) perovskites, also with an iodide to bromide ratio of 83:17. Representative *J*–*V* curves of devices with each composition are shown in Figure S14, and *t*_80_ times are shown in Figure 6a. During MPP testing, we measured PL (in a separate spectrally resolved setup) at two points: (i) in the middle of the substrate between the active area (without copper, OC condition) and (ii) on the active area (with copper, MPP condition). Bandgap trends are shown in Figure 6b. As expected, FACsI cells without bromide retain their initial bandgap of 1.54–1.55 eV throughout the whole experiment (3 weeks) with little difference between the parts with and without copper. The cells with a combination of bromide and iodide experienced a continuous drop in bandgap similar to that observed in previous experiments (50–80 meV), already after 1 day. Again, the bandgap drop occurs faster and is more prominent in the middle, between the copper stripes, where charge carriers are not extracted. Final values for all three compositions measured in the middle (dashed line) are practically the same, around 1.55 eV, while the bandgap measured on the cell area of cells with bromide (full line) is around 1.58 eV. Corresponding QFLS and PLQY tracks are shown in Supporting Information (Figure S15). Again, the bandgap shift is accompanied by the PL increase, especially in the areas without copper. The PL trends are in line with long-term stability. The more severe the bandgap change (phase segregation), the worse the stability. Bromide-free cells thus performed considerably better, retaining more than 80% of the initial power for around 700 h, on average, with the best cell exceeding 800 h. In comparison, the corresponding time for the other two compositions that contain both halide ions and experience phase segregation was 120 h in the case of FACsIBr and 80 h in the case of 3C (Figure 6a). This indicates the negative impact of phase segregation (or bromide ions) on the PSC long-term stability and that degradation of the bromide FACsIBr is due to light and not moisture, with Al_2_O_3_ capping providing a sufficient barrier for long-term stability testing in air.

**Figure 6:**
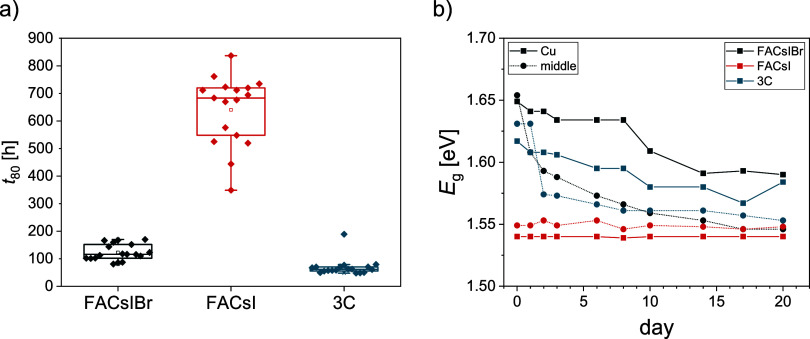
(a) *t*_80_ time from long-term MPP tracking of cells with 3 different perovskite compositions. (b) Bandgap change of the cells from the test in (a). PL was measured in the middle (dashed line) and on the active area (full line).

Since the photos in Figure 4 and measurements in Figure 6b show faster degradation when charges are not extracted (Cu vs middle), we conducted a long-term stability test with cells in different operating points: open circuit (OC), short circuit (SC, ∼0.1 V), and maximum power point (MPP) condition. Cells that were held in either OC or SC conditions were put in the MPP every day for approximately 1 h. This allowed us to monitor their performance in terms of output power, including possible stabilization time that would not occur with quick *J*–*V* measurements. We tested the two FACs compositions from above, with 17% bromide and without it.

Figure 7a presents cells that were held in either the OC or SC condition. Looking solely at their tracks (see also Figure S16 for more details), they appear stable and similar to the MPP tracks, perhaps even with less degradation. However, as shown by the daily 1 h MPP tracks (Figures 7b and S17), cells that were held in OC performed much worse than those held in SC. On average, their power fell below 80% already after 1 day of operation for FACsIBr and after 3 days for cells without bromide, despite *V*_OC_ tracks looking relatively stable. Cells of both compositions perform much better under SC conditions, retaining more than 80% of their initial power for about 300 h for cells with bromide and about 400 h for cells without Br. The devices kept in the MPP can also outperform those kept at SC,^36–38^ especially if no bromide is included. Comparison of *t*_80_ values is shown in Figure S18. Again, the performance of cells without Br is better in all three cases; however, the difference is not as large as in the previous test. We can see that degradation is much faster when the charges are not extracted from the cell and are recombined inside the perovskite (OC).^37,39^ Consequently, light soaking of the devices in OC is often used as an accelerated aging test for perovskites. However, while we see the same trend from the MPP and *V*_OC_ tracking for both perovskite compositions, the accelerating factor for the two compositions is different, and determining it will require more tests.

**Figure 7:**
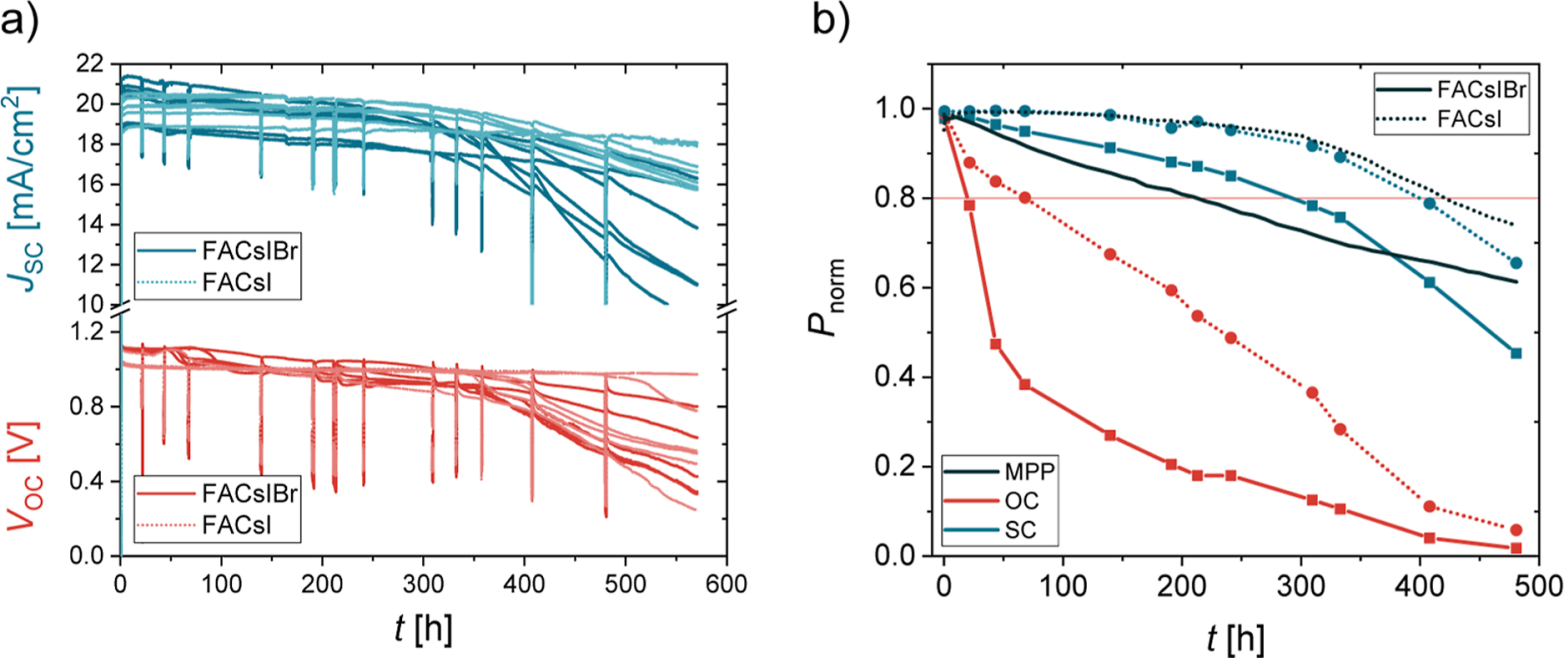
(a) *V*_OC_ and *J*_SC_ tracks of FACsIBr (darker full lines) and FACsI (lighter dotted lines) compositions. Six cells per condition were tested, 24 in total. The regular drops (e.g., at 24, 48, and 72 h) appear when the cells were put in the MPP for 1 h. Same tracks with more details and color distinction are shown in Figure S16. (b) Average values of normalized power for all cells held in a certain condition. Dots represent an average for a certain day and are connected for a better visualization of the trend.

The above operating point stability test and one of the most peculiar MPP tracks we have recorded (Figure S19) led us to further analyze the effect of operating voltage on the long-term stability. The low *V*_OC_ device in Figure S19 has stability-wise significantly outperformed the devices with much better *J*–*V* performance. Could this be connected to the voltage across the device? We selected 4 operating points with voltages of 0.6 V, 0.8 V, 1 V, and *V*_MPP_ (0.9 V) and tracked current output, while the reference cells were held in the MPP. First, the comparison between the MPP and constant voltage tracking at 0.9 V is shown in Figure S20b. After conducting three tests, no (or all of the) trends were observed; thus, we conclude that both modes yield similar results. Second, in Figure 8, we show the *t*_80_ times of all tested devices at different operating points. Interestingly, cells held at 0.6 V performed the best in terms of output current, maintaining more than 90% of the initial value for over 900 h. The second-best performing cells were those held at 0.8 V, where the current dropped below 80% after about 650 h. As expected, cells held closest to a *V*_OC_ (1 V) performed the worst, retaining 80% of the initial current for only about 150 h. Overall, we observe a clear linear correlation between the bias voltage and *t*_80_. This would imply that the SC condition would have much longer stability; however, our research and that from other groups show that MPP and SC stability are comparable. The reason behind this remains a topic for further research. Figure S20a shows the results from the second test with the same conditions and extended to 0.4 V. The same linear dependence on the bias voltage was again obtained; however, the overall *t*_80_ for that test was lower in all cases. Faster degradation at voltages above *V*_MPP_ could potentially have a significant impact on the performance of silicon-limited perovskite/Si devices, where perovskites operate at voltages above their own MPP due to not all carriers being extracted. At the same time, faster degradation at higher voltages might result in poorer long-term stability of wide-bandgap perovskite solar cells with higher *V*_MPP_. However, in both cases, more testing is needed to confirm the two hypotheses.

**Figure 8:**
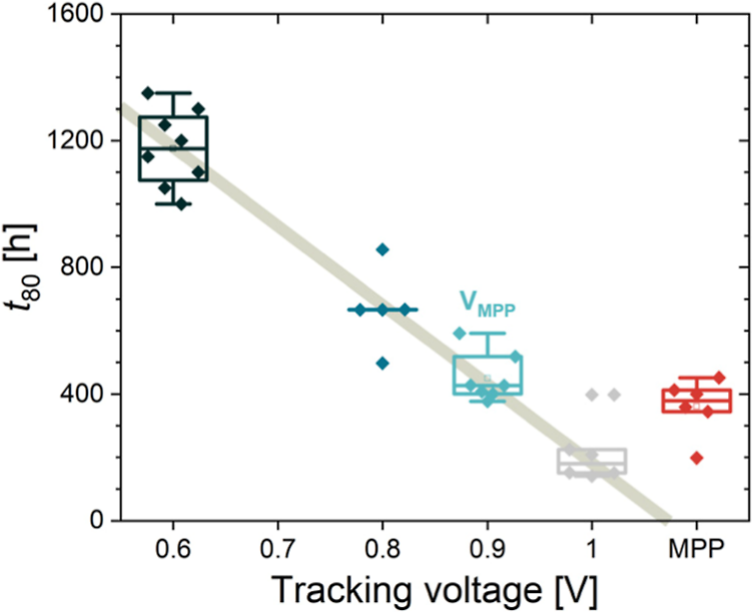
*t*_80_ time of cells held at different voltages. The *t*_80_ time for 0.6 V was extrapolated due to the WLED system failure after 900 h. The results of the repeated test are shown in Figure S20a.

Finally, in Figure 9, we compare the *t*_80_ times of reference cells tested under reference conditions (1 sun, 25 °C, MPP tracking) throughout this paper. Overall, devices were fabricated over 16 batches and exhibit significant batch-to-batch as well as intrabatch variation. The median *t*_80_ is 186 h with a standard deviation of 81 h, in stark contrast to the PCE of those devices, with a median of 16.4% and a standard deviation of 3.6%. The *t*_80_ distribution of the devices tested under the stated conditions is shown in Figure S21, while the PCE distribution is shown in Figure 1. Selecting the longest performing device could drastically alter the picture; thus, showing the stability of multiple devices should become a standard.

**Figure 9:**
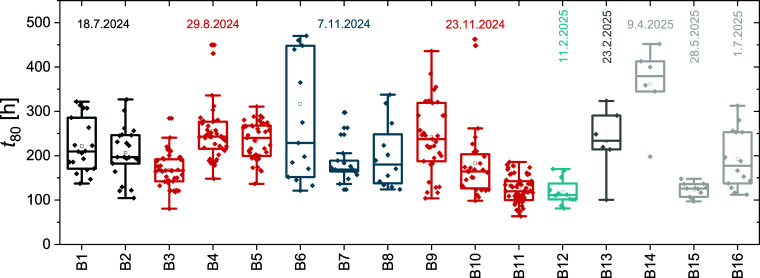
Batch-to-batch variations of reference PSCs fabricated over 16 batches and tested in different experiments, described in the paper, under reference conditions (1 sun, 25 °C, MPP tracking). Dates represent the start of the experiment, while the same colors represent the same type of experiment: Black: Figure 3a, red: Figure 2a, blue: Figure 3b, light blue: Figure 6a, gray: Figure 7b, light gray: Figure 8.

## Conclusions

In this work, we systematically investigated the influence of humidity and light on the stability of a perovskite solar cell. By performing water and damp heat tests, we showed that a 30 nm thick Al_2_O_3_ capping layer sufficiently blocks moisture and enables long-term indoor measurements in air without the need for encapsulation. Instead, most of the degradation during operation is caused by light.

To evaluate this, we developed a white LED long-term stability setup in which we can set and test different operating conditions and used *t*_80_ time as a degradation metric. The cells were first tested under continuous and cyclic illumination, followed by a test under different light intensities. In both experiments, we found an important role of light in cell degradation. The *t*_80_ time under cyclic illumination doubled, corresponding to the time the cells were exposed to light; thus, the stability is practically the same. This indicated no recovery during the dark part of the cycle. Under lower light intensities, the *t*_80_ was longer; however, we do not see a linear trend between lifetime and light intensity.

During testing, we have observed severe visual degradation of the films. Several degradation areas and modes can be seen; however, in general, the degradation was stronger in inactive regions and has also expressed itself in a bandgap red shift. Continuous long-term PL monitoring confirmed slow red shifting of the bandgap, PL increase, and degradation at the perovskite/C_60_ interface. To avoid phase segregation, devices without bromide were tested. Indeed, the stability of the devices without bromide improved 5-fold in the best case; however, the perovskite/C_60_ remained critical.

Finally, by tracking the devices under different operating conditions, we determine the (bias) voltage across the device as one of the main causes of degradation. The devices degrade the fastest under open-circuit conditions, while by reducing the operating voltage toward 0.4 V, we observe a linear increase in the *t*_80_ time.

In total, more than 500 devices were tested, and each test consisted of devices from several batches. Despite the high reproducibility of PCE results, we observe intrabatch and especially batch-to-batch variations in stability *t*_80_ time, ranging from below 100 to more than 500 h. This highlights that for relevant stability results, numerous devices have to be not only tested but also reported, as has been the case in our case. Only then can clear and objective conclusions be drawn.

## Experimental Section

### Device Fabrication

Perovskite solar cells analyzed in this paper utilize an inverted planar structure and have a layer configuration of glass/ITO/MeO-2PACz/perovskite/C_60_/SnO_2_/Cu/AlOx. As a substrate, a patterned ITO-coated glass (Automatic research, *R* = 15 Ω sq^–1^) accommodating 6 solar cells is used, with the end size of individual solar cell being about 0.17 cm^2^.

The substrates are first cleaned in an ultrasonic bath in a four-stage process: acetone, mucasol (2%), deionized water, and isopropanol, 12 min each time. Between the cleaning steps, the samples are thoroughly rinsed with deionized water. The cleaning process is finished with a 15 min cleaning with UV light and ozone, which removes any remaining organic material and improves the wettability of the substrates.

The FACs (FA_0.83_Cs_0.17_Pb­(I_0.83_Br_0.17_)_3_) perovskite solution is prepared by mixing stock solutions of PbI_2_, PbBr_2_, and CsI with FAI powder. All stock solutions are prepared with a molarity of 1.5 M. Solutions of PbI_2_ and PbBr_2_ are made by dissolving the respective salts in DMF/DMSO (4/1) solvent, while CsI is dissolved in pure DMSO. For the hole transport layer (HTL), a monolayer MeO-2PACz is used, which is prepared by dissolution of 1 mg in 4 mL of ethanol.

Application of layers starts with spin coating of MeO-2PACz at 3000 rpm for 30 s, with an initial 5 s acceleration. Substrates are then annealed at 100 °C for 10 min. Next, the layer of perovskite is spin-coated at 4000 rpm for 35 s with an initial 5 s acceleration, followed by another step of annealing at 100 °C for approximately 35 min. Twenty-five seconds after the start of spin coating, the films are rinsed with ethyl acetate as an antisolvent. A layer of C_60_, which serves as an ETL, is deposited in the evaporator at a rate of around 0.1 Å/s with a final thickness of 21 nm.

SnO_2_ and Al_2_O_3_ layers are deposited by the ALD tool with high thickness precision. Twenty nm (140 cycles) of SnO_2_ was deposited at 80 °C by alternatingly depositing H_2_O and TDMASn (Tetrakis­(Dimethylamino)­Tin) precursors. The thickness of the Al_2_O_3_ capping layer is around 30 nm. The process is carried out at 100 °C using H_2_O and TMA (trimethylamine) precursors. After SnO_2_ and before Al_2_O_3_, 100 nm of copper is evaporated as the top electrode.

Before spraying with a PCB plastic spray, contacts of fabricated devices are protected by office tape to prevent an insulating layer of spray. The tape was removed after spraying. Cells were allowed to dry completely before further testing.

### Characterization

#### Current–Voltage (*I*–*V*)

The current–voltage curve was measured using a Keithley 2400 Source Meter Unit in air under the illumination of simulated AM 1.5 G solar light from a Newport solar simulator system, class ABA. The scan rate was 0.25 V s^–1^ with a step of 20 mV.

#### Damp Heat

Damp heat experiments were performed in an environmental chamber from Memmert. Cells were placed on a plastic tray with a glass side facing down. They were taken from the chamber regularly to measure their *J*–*V* curves and PL response. During that time, cells were exposed to ambient conditions for approximately 1–2 h.

#### Stability Testing

Stability testing was performed using a custom-built WLED setup. Illumination is provided by white LEDs (1-sun equivalent intensity), and the samples were kept at 25 °C. The devices (up to 144, 24 substrates with 6 devices each) were tracked using a custom-built MPP tracking system. All measurements were performed in air. A photo of the system is shown in Figure S2b.

#### Photoluminescence

Photoluminescence was measured using the LuQY system from QYB (Quantum Yield Berlin). It automatically determines the PLQY, bandgap, and QFLS of the tested films or devices. For excitation, a laser with a wavelength of 532 nm was used, while spectra were recorded with an integrating sphere and spectrometer. Integration times ranged from 100 ms to 4 s. All results were time-normalized.

#### Long-Term PL Tracking

Long-term PL tracking was performed under 1-sun equivalent illumination with a blue LED matrix with a peak wavelength of 470 nm. Images were captured every 5 min at a 45° angle using a Thorlabs LP126MU 12MP monochrome CMOS camera with a later perspective correction. To avoid saturation, each image was captured at three different exposure times (10, 50, and 100 ms) and normalized to exposure time. Unsaturated counts from the active area were integrated to show the PL intensity in Figure S11.

## Supplementary Material



## Notes

Detailed description of water and damp-heat tests, statistics of *J*–*V* measurements, time-dependent MPP and PL tracks from various experiments, and additional batch-to-batch analysis (PDF)
