# Supplementary material for: 500+ FACs Perovskite Solar Cells under Long-Term Stability Testing
Source: ACS Appl Mater Interfaces. 2026 Jan 7;18(2):3946–55. doi: 10.1021/acsami.5c24756 (PMC12828718; doi:10.1021/acsami.5c24756)
Supplement: Supplementary file 1 [file am5c24756_si_001.pdf]

Supporting information

500+ FACs perovskite solar cells under long-term stability testing

Žan Ajdič[1], Fernando Solorio Soto[1], Marko Remec[1, 2], Miha Kikelj[1], Arslan Ali[1], Boštjan Glažar[1], Gašper Matič[1], Kristijan Brecl[1], Marko Jankovec[1], Marko Topič[1], Marko Jošt[1, *]

[1]University of Ljubljana, Faculty of Electrical Engineering, Tržaška 25, 1000 Ljubljana, Slovenia

[2]Helmholtz-Zentrum Berlin für Materialien und Energie, 12489 Berlin, Germany

*Corresponding author: marko.jost@fe.uni-lj.si

Reference solar cells and stability setup

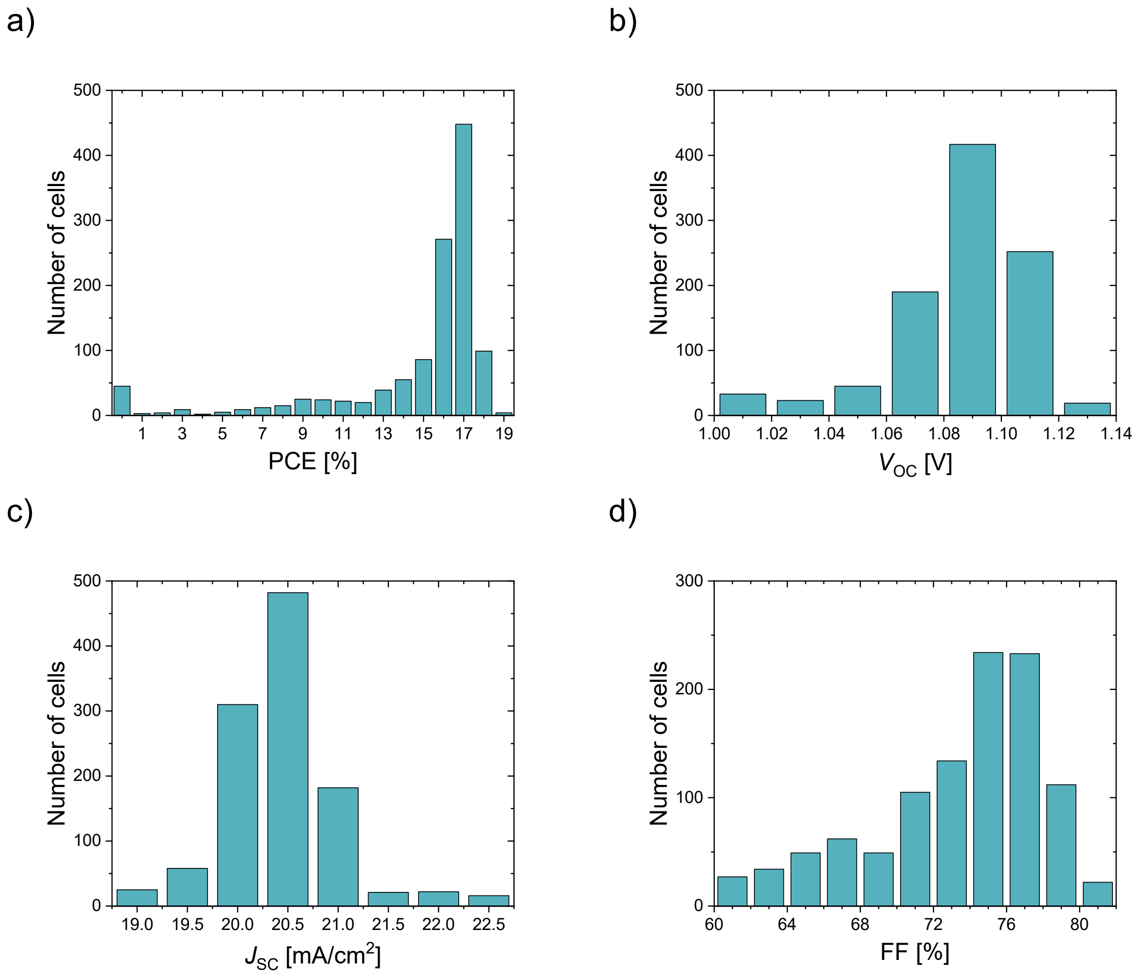

Figure S1: PV performance parameters of all reference cells used in experiments for this paper. a) PCE_{mean} = 15.1%, PCE_{median} = 16.4%. b) $V_{OC,mean}$ = 1.05 V, $V_{OC,median}$ = 1.09 V. c) $J_{SC,mean}$ = 19.9 mA/cm^2, $J_{SC,median}$ = 20.3 mA/cm^2. d) FF_{mean} = 72%, FF_{median} = 74.5%.

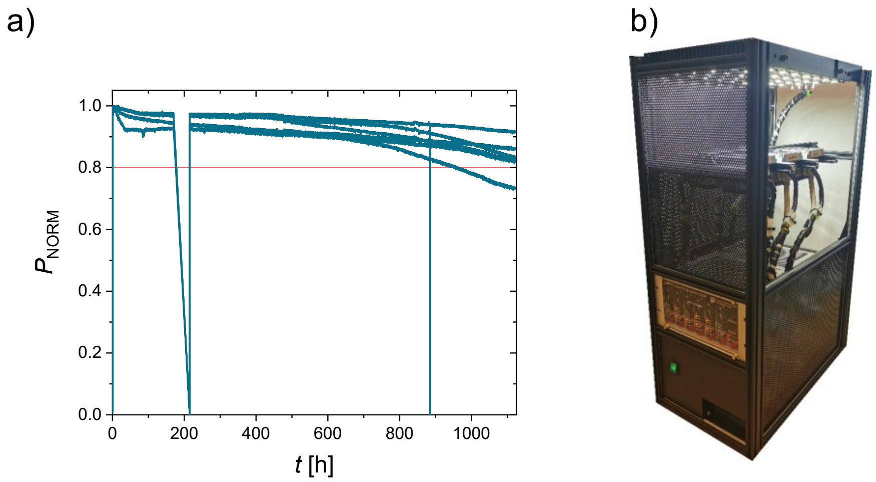

Figure S2: a) MPP track of the most stable reference device. b) Photo of the in-house made WLED setup with white LED light source and 144 MPP tracking channels that was used for stability testing.

Supporting Note 1

Water and damp-heat tests

Water test

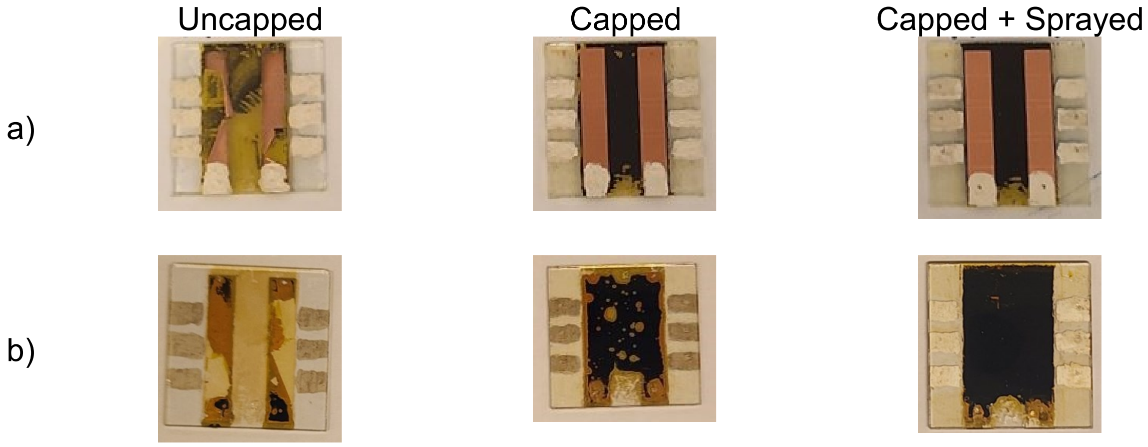

Figure S3: Photos of immersed cells a) after 15 minutes and b) after 2 hours in water.

For the water test, we submerged perovskite substrates in water for a total of 2 hours and measured their performance (*J-V* curve) every 15 min under the sun simulator. We compared 3 different cases: i) unprotected, uncapped device, ii) Al_2O_3 capped device, and iii) Al_2O_3 capped and sprayed device. After the first hour, uncapped cells without any protection visually almost completely degraded, while the other two combinations remained relatively intact (Figure S3a). The *J-V* measurements confirmed total failure of the uncapped cells with their PCE dropping to 0%. For the capped cells, one could still see a lot of black areas at the end of the experiment (after 2 hours), indicating non-degraded perovskite (Figure S3b). Nevertheless, their *J-V* performance exhibited a steady decrease, reaching 5% at the end of the test (Figure S4). The best result was obtained in the case with a combination of both Al_2O_3 capping and PCB spray. *J-V* measurements show almost no drop for all PV performance parameters under STC after the water test, confirming the formation of an efficient water barrier (Figure S4). Same trends were obtained from a 3 hour water test with measurements every hour (not shown here).

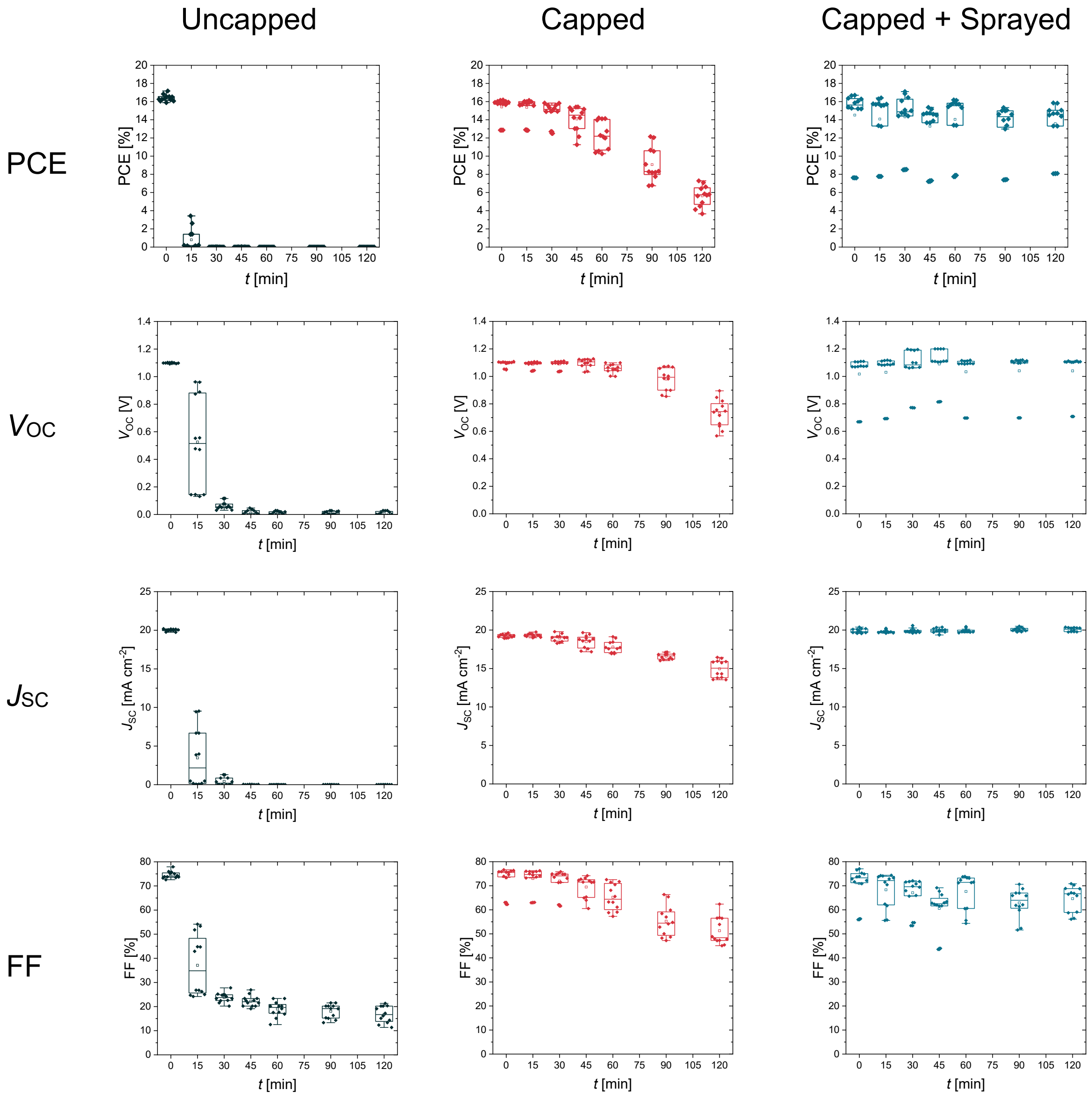

Figure S4: PCE, V_{OC}, J_{SC} and FF parameters of the cells from the water test measured every 15 min. Three types of devices were tested: uncapped, capped with Al_2O_3, and capped and sprayed.

Damp-heat test

The moisture resilience was also evaluated with damp-heat tests. The initial tests were performed at 80 °C and 80% relative humidity. Selected conditions caused a very fast degradation of our non-encapsulated devices, therefore we decided to reduce the stress factors by lowering both the temperature and relative humidity for better comparison of the degradation between different protective methods.

The capped and uncapped cells were then tested at three different temperatures (25, 50 and 60 °C) and 60% relative humidity. We periodically measured the PL response as well as *J-V* curves at STC. In all cases, PCE shows a burn-in effect during the first 2 days. Afterwards, uncapped devices start degrading at an accelerated rate, especially at higher temperatures, while capped devices perform much better. For

example, uncapped devices at 50 °C lost almost half of the initial PCE in the first day while for capped cells this happens around day 10. As expected, the capped solar cells tested at 25 °C performed best. After the initial burn-in, the PCE was stable until day 17. Similar trends can be observed in the PL measurements, where we were most interested in tracking the bandgap shift (Figure S5b). The bandgap decrease of 40-60 meV is similar in all cases, however, it occurs faster with uncapped devices at higher temperatures. The exception is the capped device at room temperature, where the bandgap drop is negligible, while in the case of the uncapped cell it is only delayed. The red shift in perovskite bandgap is typically associated with phase segregation under light.[35, 36] Here we observe similar effects under elevated temperatures and humidity.

The comparison between capped devices with and without spray during 50 °C/60% damp heat testing is shown in Figure S6. In this case, the capped devices performed slightly better in comparison to devices with additional sprayed layer.

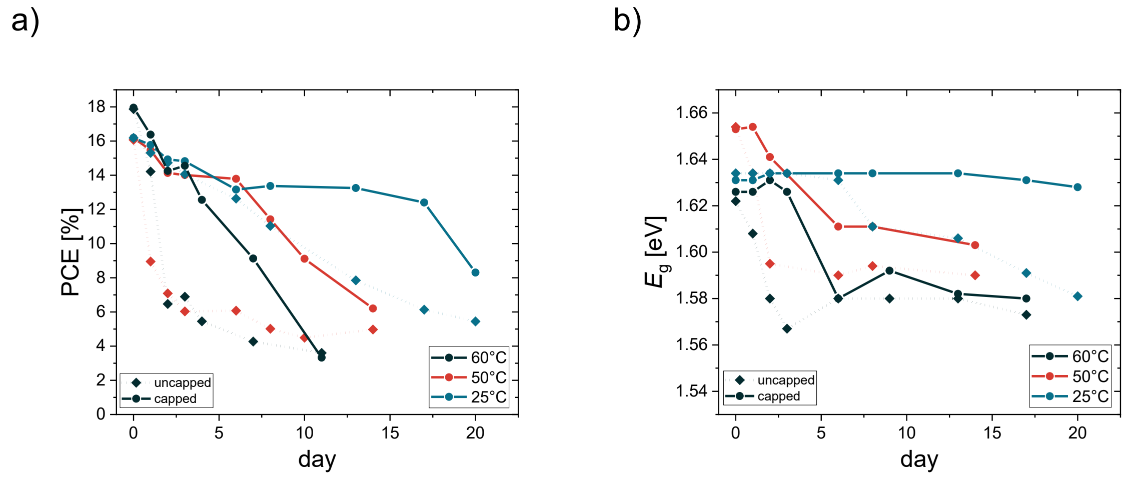

Figure S5: a) Average PCE of 12 solar cells in each combination. Test at 60 °C ran for 17 days, test at 50 °C for 14 days and test at 25 °C for 20 days, b) Bandgap change of PSCs during time for cells at the three different temperatures and 60% relative humidity.

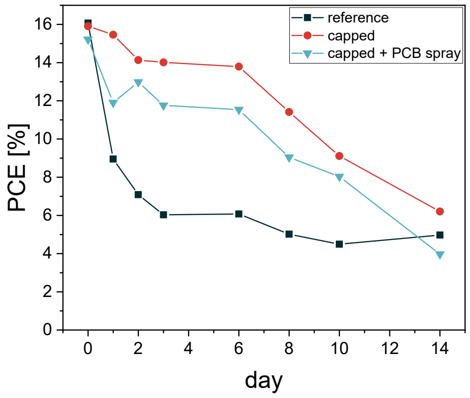

Figure S6: The comparison between uncapped devices, capped devices and capped devices with PCB spray during 50 °C/60% damp heat testing.

Effect of PCB spray and typical MPP tracks

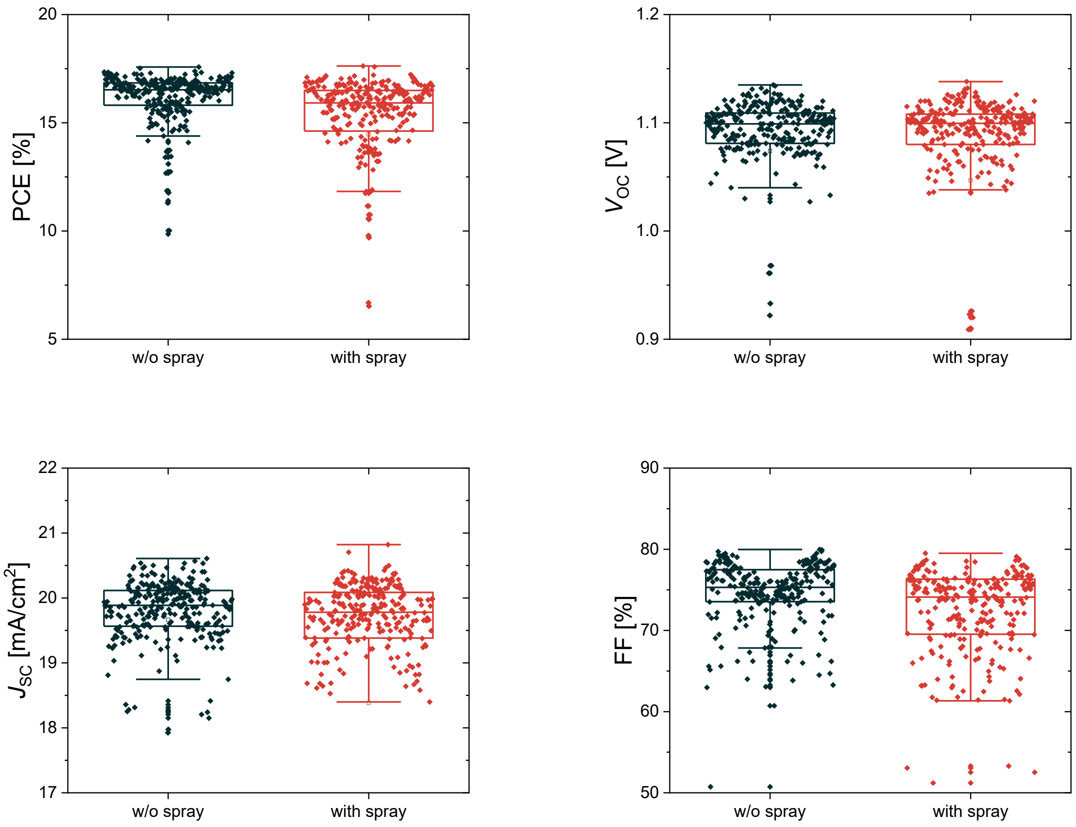

Figure S7: PV performance parameters of devices with Al_2O_3 capping (black) and with Al_2O_3 capping and PCB spray (red).

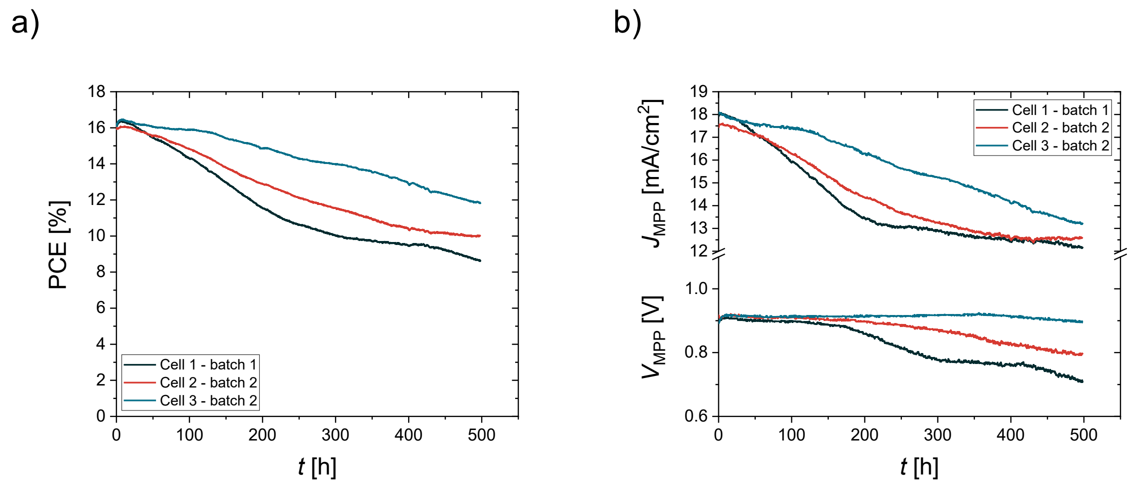

Figure S8: a) Long-term P_{MPP} of selected cells from Figure 2a. b) J_{MPP} and V_{MPP} of the same cells as on the left.

Light intensity tests

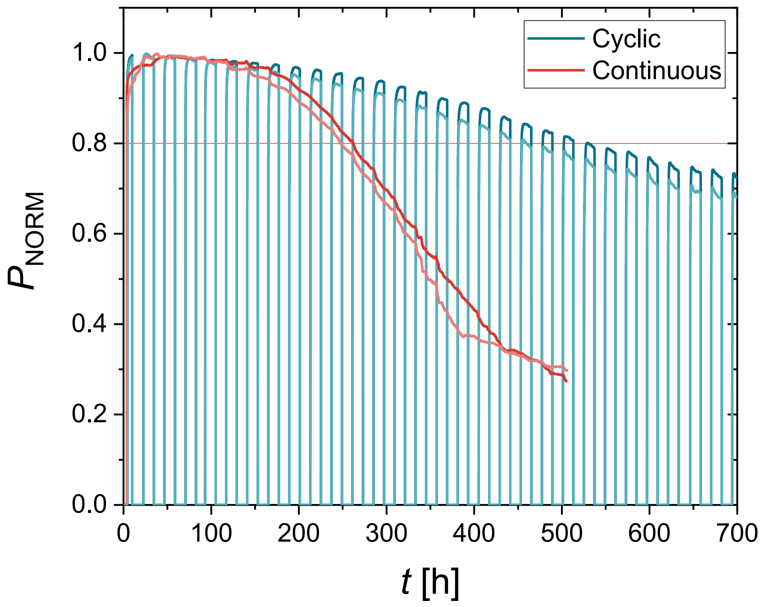

Figure S9: Comparison of selected cells under continuous and cyclic light. Normalized P_{MPP} track of 2 cells under each condition are shown.

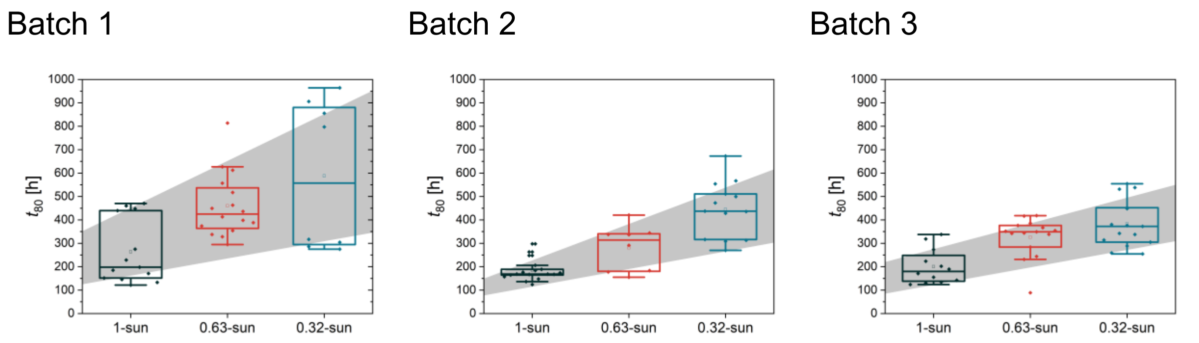

Figure S10: t_{80} for cells fabricated in different batches and tested under different illumination intensities (1-sun without filter, 0.63-sun with OD02 filter and 0.32-sun with OD05 filter). The graph combining all three graphs is shown in the main part in Figure 3b.

Long-term PL tracking

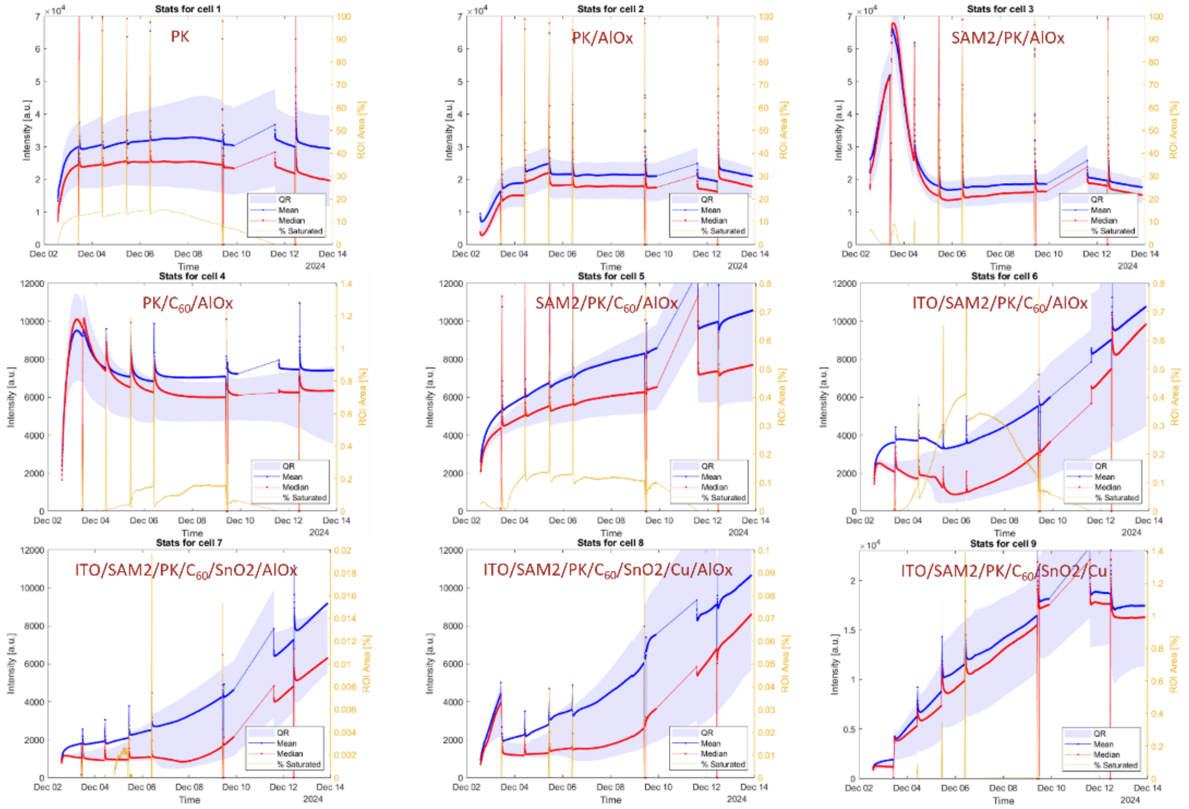

Figure S11: PL intensity for 9 different layer stacks (from only perovskite to full stack as labelled) during long-term PL tracking. The values on the y-axis are integrated counts from the PL images shown in Figure 5.

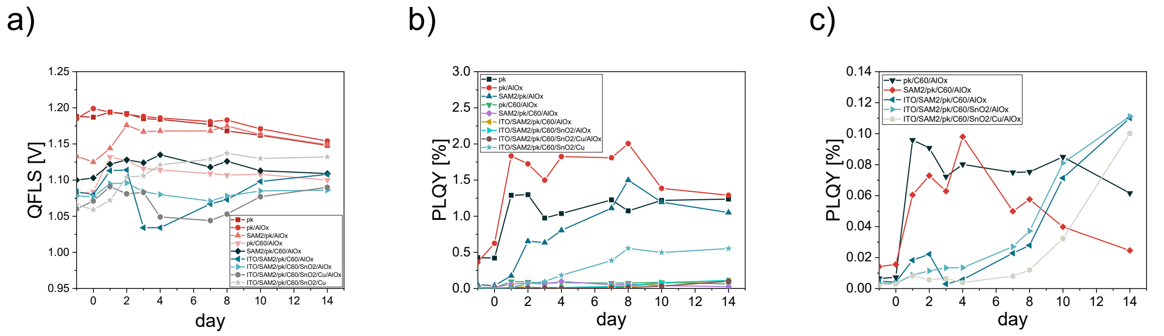

Figure S12: a) QFLS and b) PLQY of samples shown in Figure 5 and Figure S11, measured with a spectrally resolved PL system. c) Zoom in of PLQY of films from b).

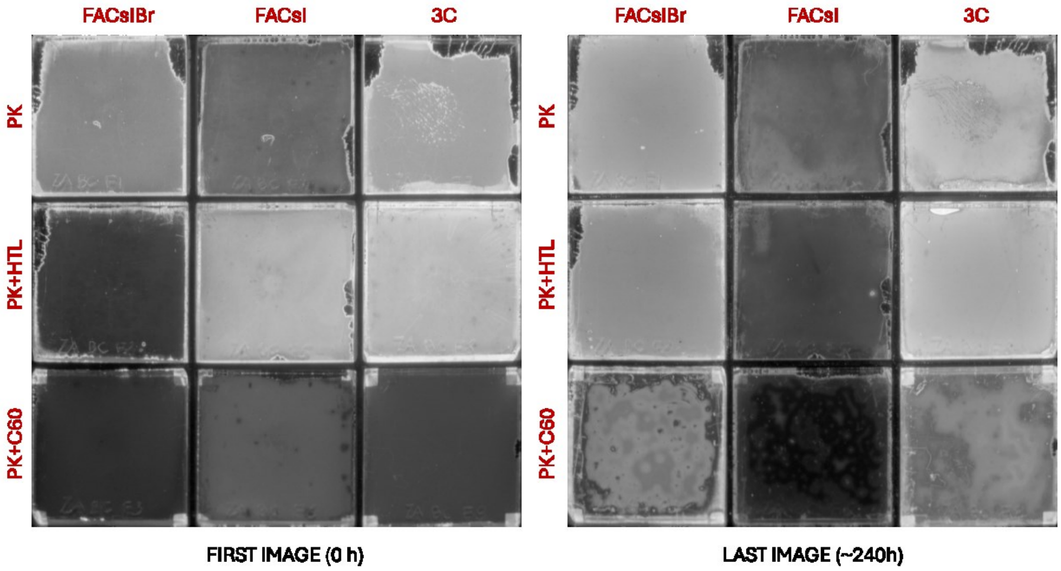

Figure S13: PL image of fresh films (left) and aged films for 10 days (right) taken with a camera. 3 different compositions and 3 layer stacks were tested as marked on picture. Layer stacks containing C_{60} (third row) degraded much more than films without it, as can be concluded from the increased inhomogeneity.

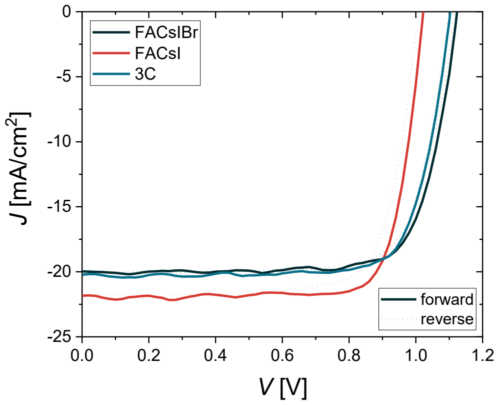

Figure S14: Representative J-V curves from each composition composition used in the paper.

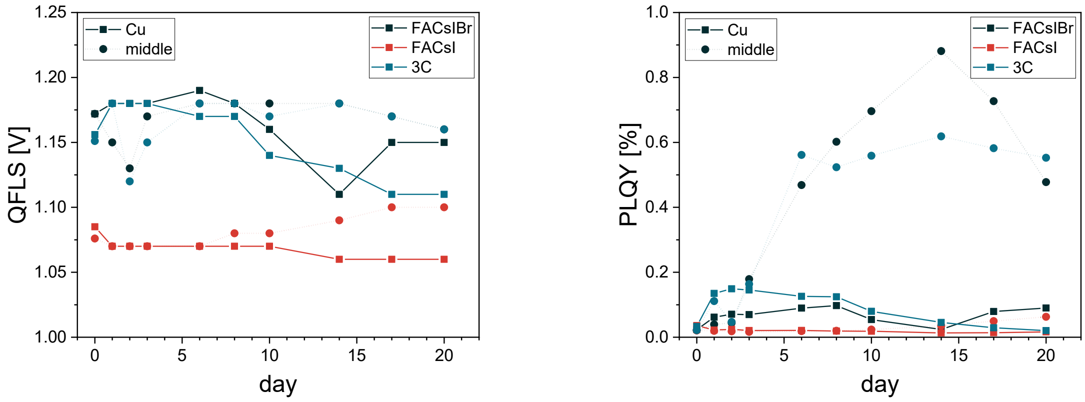

Figure S15: Regular QFLS and PLQY measurements of cells with different compositions during long-term MPP tracking. The MPP tracking results and bandgap changes are shown in Figure 6.

Influence of operating point conditions

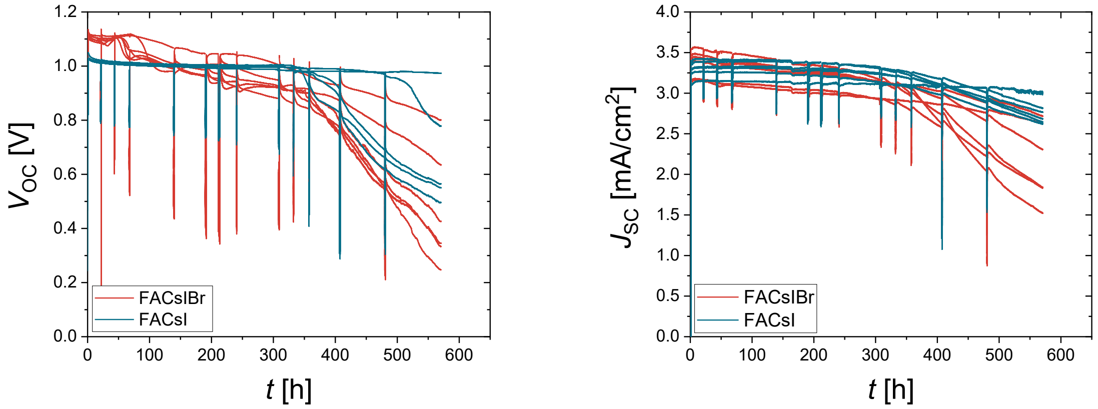

Figure S16: Long-term a) V_{OC} and b) J_{SC} tracking. Same tracks as in Figure 7a, but with more details and color distinction. The devices were regularly (daily) put to the MPP for one hour as can be seen by the regular drops in the tracks. The MPP tracks are shown in Figure S17.

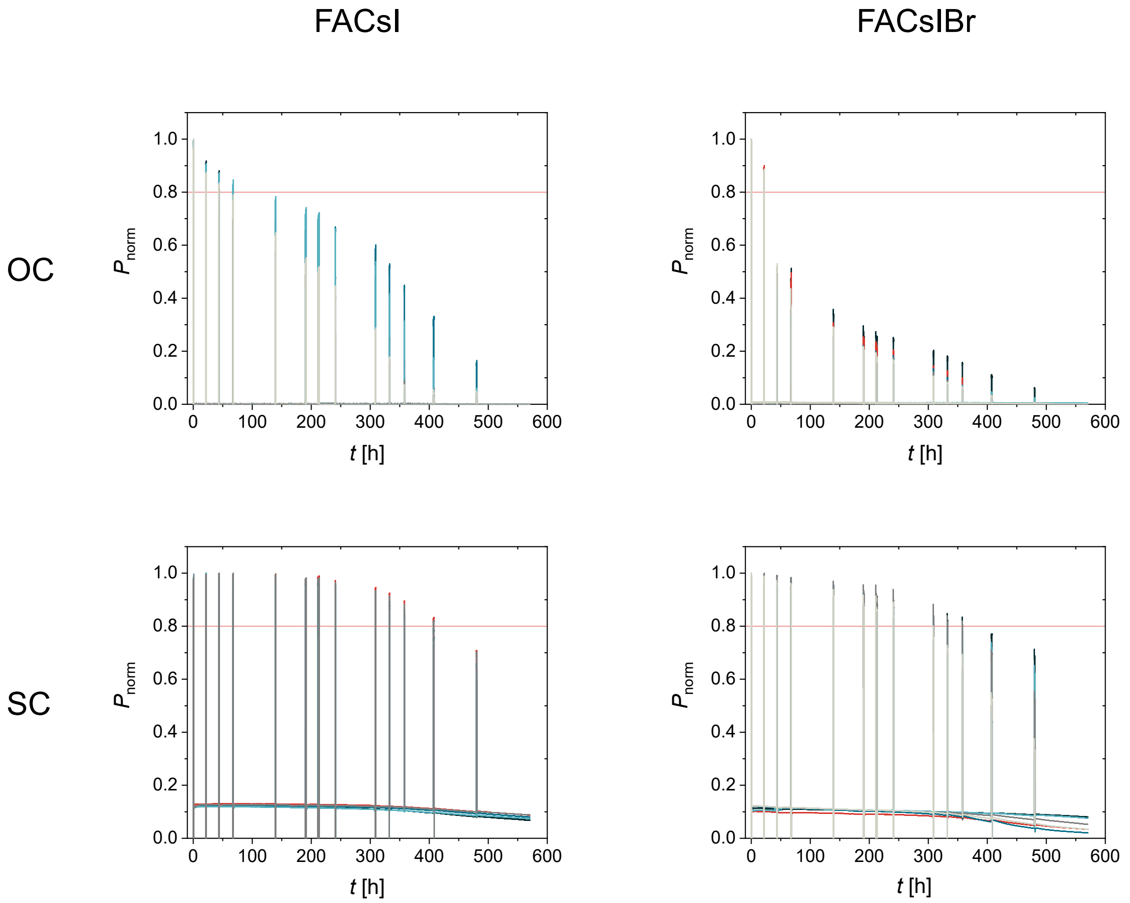

Figure S17: Regular MPP tracks during long-term V_{OC} and J_{SC} tracking. First row represents hourly MPP tracks of cells with (right) and without (left) bromide held at OC, while second row shows the same for cells held at SC.

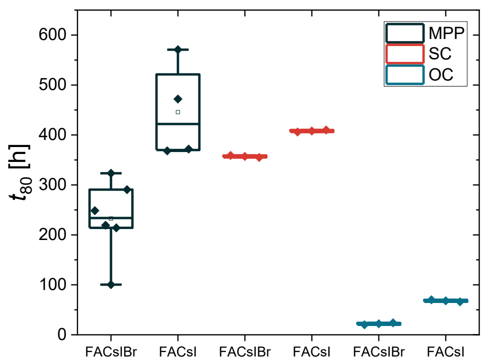

Figure S18: t_{80} for FACsIBr and FACsI cells held at certain operating point condition (maximum power point, open-circuit and short-circuit).

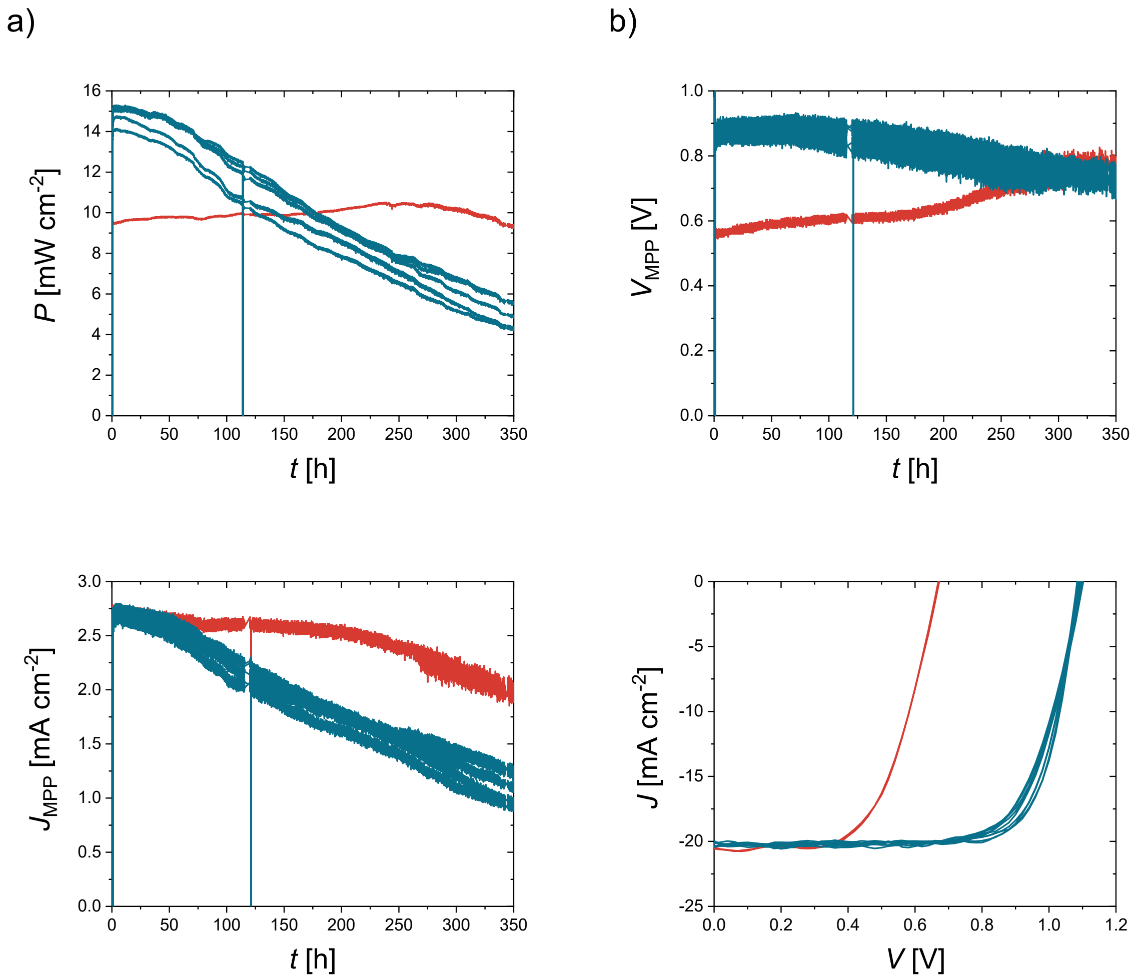

Figure S19: An example of a MPP track of 6 cells from the same substrate: a) P, b) V_{MPP} and c) J_{MPP} are shown. d) The J-V curves of the same cells. The cell with a better stability (red lines) is the cell with much lower V_{OC} and is therefore tracked at lower V_{MPP}.

a) b)

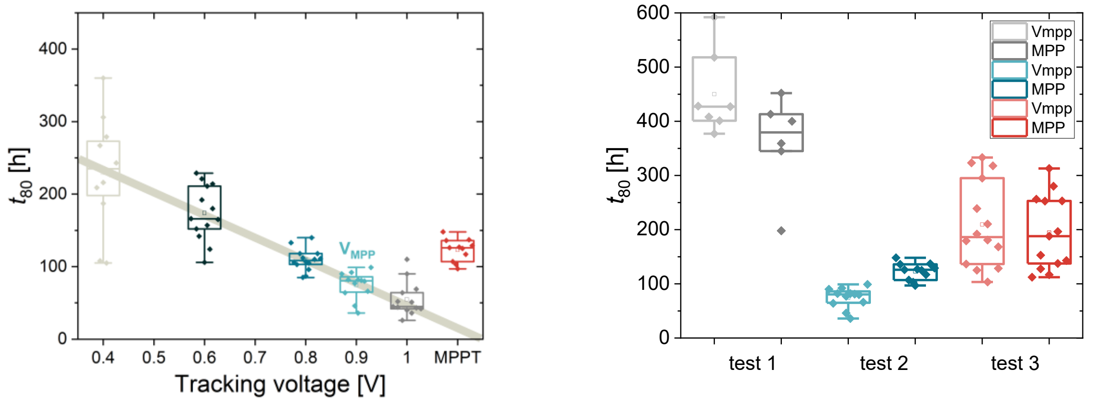

Figure S20: a) Stability of PSCs held at different constant voltages (second test). b) t_{80} of cells kept at a constant V_{MPP} voltage (~0.9 V) and those in MPPT mode from 3 different tests.

Batch-to-batch variations

a) b)

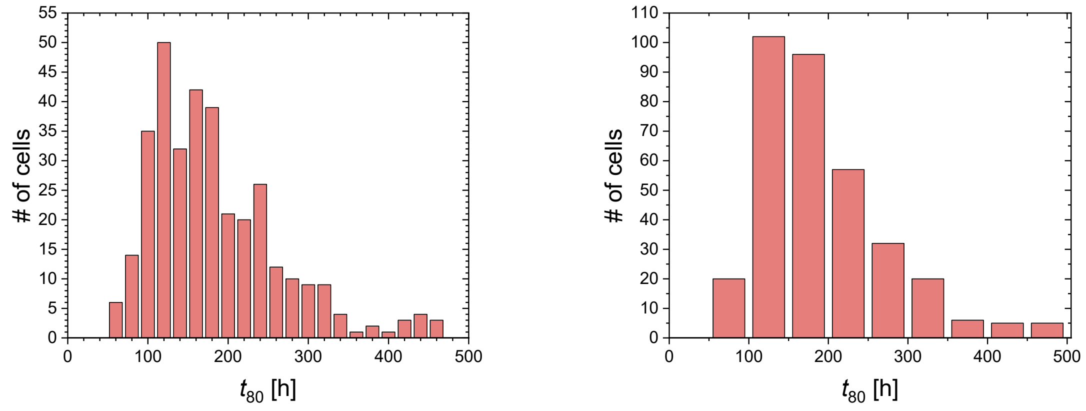

Figure S21: Distribution of stability t_{80} time of the reference cells tested under reference conditions (1-sun, 25 °C), fabricated over multiple batches as shown in Figure 9 in the main part. t_{80} binned in the groups of a) 20 hours and b) of 50 hours. t_{80_mean} = 198 h, t_{80_median} = 186 h and with a standard deviation of 81 h.
